# Photoinduced Oxygen Evolution Catalysis Promoted by Polyoxometalate Salts of Cationic Photosensitizers

**DOI:** 10.3389/fchem.2018.00302

**Published:** 2018-08-14

**Authors:** Joaquín Soriano-López, Fangyuan Song, Greta R. Patzke, J. R. Galan-Mascaros

**Affiliations:** ^1^Institute of Chemical Research of Catalonia, Barcelona Institute of Science and Technology, Tarragona, Spain; ^2^Departament de Química Física i Inorgànica, Universitat Rovira i Virgili, Tarragona, Spain; ^3^Department of Chemistry, University of Zurich, Zurich, Switzerland; ^4^ICREA, Passeig Lluis Companys, Barcelona, Spain

**Keywords:** water splitting, oxygen evolution, polyoxometalates, photosensitizer, cobalt

## Abstract

The insoluble salt Cs_15_K[Co_9_(H_2_O)_6_(OH)_3_(HPO_4_)_2_(PW_9_O_34_)_3_] (**CsCo**_9_) is tested as heterogeneous oxygen evolution catalyst in light-induced experiments, when combined with the homogeneous photosensitizer [Ru(bpy)_3_]^2+^ and the oxidant Na_2_S_2_O_8_ in neutral pH. Oxygen evolution occurs in parallel to a solid transformation. Post-catalytic essays indicate that the **CsCo**_9_ salt is transformed into the corresponding [Ru(bpy)_3_]^2+^ salt, upon cesium loss. Remarkably, analogous photoactivated oxygen evolution experiments starting with the [Ru(bpy)_3_]_(5+x)_K_(6−2x)_[Co_9_(H_2_O)_6_(OH)_3_(HPO_4_)_2_(PW_9_O_34_)_3_]·(39+x)H_2_O (**RuCo**_9_) salt demonstrate much higher efficiency and kinetics. The origin of this improved performance is at the cation-anion, photosensitizer-catalyst pairing in the solid state. This is beneficial for the electron transfer event, and for the long-term stability of the photosensitizer. The latter was confirmed as the limiting process during these oxygen evolution reactions, with the polyoxometalate catalyst exhibiting robust performance in multiple cycles, upon addition of photosensitizer, and/or oxidant to the reaction mixture.

## Introduction

Sunlight is the preferred carbon-neutral energy source for competing with fossil fuels for energy production, because solar radiation is readily accessible at almost any location on the surface of the Earth (Cook et al., 2010). Artificial photosynthesis aims to mimic natural photosynthesis, where sunlight is stored in the form of chemical bonds through reduction of CO_2_ into sugars, employing H_2_O as the ultimate source of electrons (Mcevoy and Brudvig, 2006). Therefore, an artificial photosynthesis device would convert sunlight into spatially separated electron/hole pairs and store its energy subsequently into chemical bonds by means of water splitting, obtaining hydrogen as a clean fuel together with oxygen as the only side product (Lewis and Nocera, 2006; Balzani et al., 2008; Barber, 2009). Unfortunately, the market introduction of commercial artificial photosynthesis devices is still hampered by the lack of robust, inexpensive and efficient water oxidation catalysts (WOCs) (Dau et al., 2010; Seh et al., 2017).

Over the last decades, scientists have reported a wide variety of new WOCs. Homogeneous organometallic compounds work at fast oxygen evolution rates and offer good processability (Concepcion et al., 2008, 2009; Bozoglian et al., 2009; Blakemore et al., 2010; Xu et al., 2010; Lloret-Fillol et al., 2011; Mccool et al., 2011; Barnett et al., 2012; Duan et al., 2012; Liu and Wang, 2012; Zhang et al., 2013; Goberna-Ferrón et al., 2014). However, they often suffer from limited long-term stability due to oxidative degradation of the organic ligands in the harsh working conditions needed for water oxidation. Precious-metal-based WOCs, for instance Ir-, and Ru-based materials, have shown superior performance and stability for water oxidation catalysis (Pillai et al., 2000; Youngblood et al., 2009; Blakemore et al., 2010; Duan et al., 2012). Unfortunately, the high production price due to metal scarcity questions their viable implementation into commercial devices. Earth abundant transition metal oxides and perovskites are a robust alternative, but exclusively in alkaline media (Galán-Mascarós, 2015). Therefore, alternatives to the current state-of-the-art catalysts are needed.

Polyoxometalates (POMs) have recently appeared as a promising new catalyst class (Geletii et al., 2008; Sartorel et al., 2008). When employed as WOCs, they combine the most appealing features of homogeneous and heterogeneous materials, and many of them can be obtained from inexpensive raw materials. They are all-inorganic molecular clusters with high stability under strongly oxidizing conditions. At the same time, their molecular nature provides access to the tunability and superior processing capabilities of homogeneous catalysts for their easier implementation into devices (Pope, 1983; Pope and Müller, 2001). POMs have shown high catalytic activity in water oxidation over a remarkable pH range (0-10), and they retain their catalytic activity under heterogeneous conditions as their corresponding insoluble salts, or when anchored onto solid supports (Wu et al., 2012; Guo et al., 2013; Quintana et al., 2013; Soriano-López et al., 2013).

**Figure 1:**
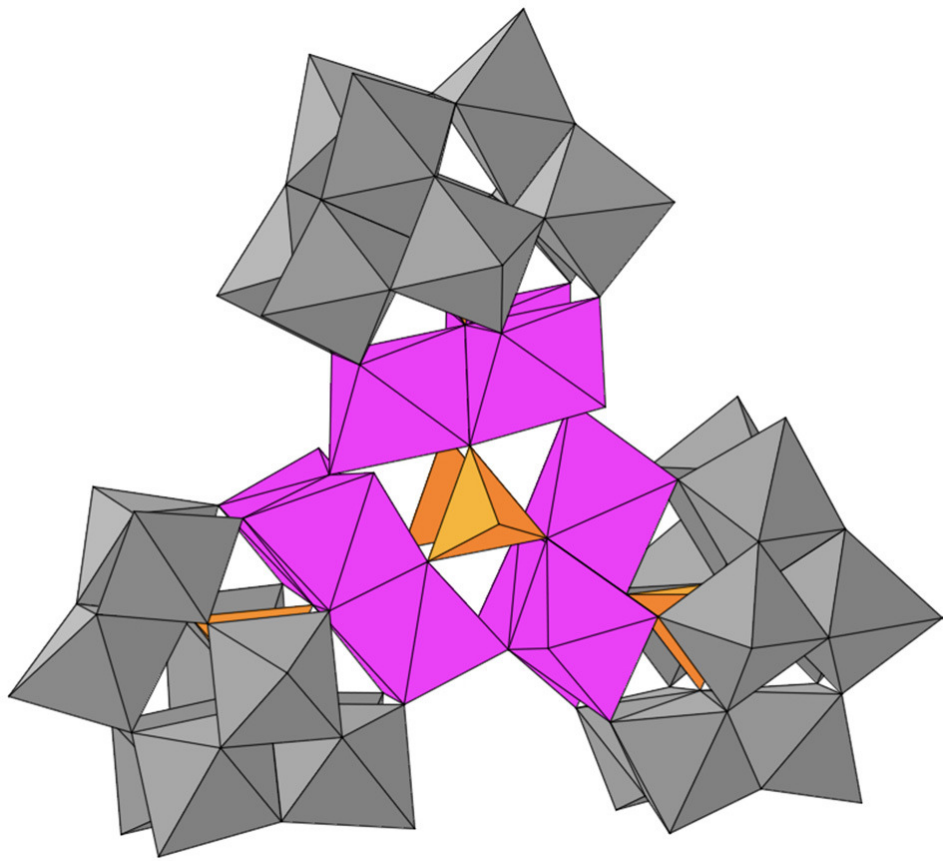
Polyhedral representation of the polyanion [Co_9_(H_2_O)_6_(OH)_3_(HPO_4_)_2_(PW_9_O_34_)_3_]^16−^ (**Co_9_**); WO_6_, gray octahedra; PO_4_, orange tetrahedra; CoO_6_, pink octahedra.

Among polyoxometalates, the cobalt-containing POMs (Co-POMs) have emerged as the most promising WOCs due to their high efficiency and kinetics (Goberna-Ferrón et al., 2012; Lv et al., 2012, 2014; Evangelisti et al., 2013). After Hill et al. reported the OER activity of the [Co_4_(H_2_O)_2_(PW_9_O_34_)_2_]^10−^ polyanion (Yin et al., 2010; Huang et al., 2011; Stracke and Finke, 2011, 2013, 2014), we turned our attention to the high nuclearity [Co_9_(H_2_O)_6_(OH)_3_(HPO_4_)_2_(PW_9_O_34_)_3_]^16−^ (Goberna-Ferrón et al., 2012, 2015; Soriano-López et al., 2013; Co_9_, Figure 1). **Co**_9_ shows good activity for photo-assisted water oxidation in homogeneous conditions, exhibiting fast charge transfer kinetics with the model [Ru(bpy)_3_]^2+^ photosensitizer (bpy = 1,2-dipyridyl; Natali et al., 2017). It is also active in the solid state when processed as an insoluble salt with alkaline metal countercations (Soriano-López et al., 2013).

In this work we report the next required step on the road to technological applications for **Co_9_** in an artificial photosynthesis platform, namely its combination with a photosensitizer in a light-induced process in heterogeneous conditions. These essays have been very successful with other POMs and water oxidation catalysts to assess photo-induced catalytic performance, mechanistic considerations, and stability issues (Puntoriero et al., 2010; Gao et al., 2013; Sartorel et al., 2013; Al-Oweini et al., 2014; Xiang et al., 2014; Natali et al., 2017). Our experiments confirm the efficient electron transfer between catalyst and sensitizer, even when both species are combined into an insoluble salt. The latter opens up interesting possibilities for future combinations of cationic photosensitizers with polyanionic WOCs for the construction of compact functional photoelectrodes.

## Experimental section

### Materials and synthesis

Tris(2,2′-bipyridyl)dichlororuthenium(II) hexahydrate and sodium persulfate were purchased from TCI and Sigma-Aldrich (>99% purity) and used without further purification. The synthesis of Cs_15_K[Co_9_(H_2_O)_6_(OH)_3_(HPO_4_)_2_(PW_9_O_34_)_3_]·39H_2_O (**CsCo_9_**) was already reported (Soriano-López et al., 2013). [Ru(bpy)_3_]_(5+x)_K_(6−2x)_[Co_9_(H_2_O)_6_(OH)_3_(HPO_4_)_2_(PW_9_O_34_)_3_]·(39+x)H_2_O (**RuCo_9_**) was prepared by metathesis: A stoichiometric excess of [Ru(bpy)_3_]Cl_2_ was added to a solution containing Na_8_K_8_[Co_9_(H_2_O)_6_(OH)_3_(HPO_4_)_2_(PW_9_O_34_)_3_]·43H_2_O (**KCo_9_**). **RuCo_9_** immediately precipitated as an orange powder. It was filtered, washed with water and acetone, and air-dried.

### Material characterizations

Elemental CHN analysis was performed with an Elemental Microanalyzer Flash model 1112. Detection of Co, Ru, and W was performed on an inductively coupled plasma atomic emission spectrometer iCap 6500 (Thermo Fisher Scientific), and K was detected on a 2,380 atomic absorption spectrophotometer (Perkin-Elmer), both by Mikroanalytisches Labor Pascher (Remagen/Germany). Thermogravimetric analyses were performed with powder samples using a TGA/SDTA851 Mettler Toledo with a MT1 microbalance. Dynamic light scattering was used to measure the particle size distribution employing a Malvern NanoZS analyzer. FT-IR spectra were collected in the 3600–400 cm^−1^ range with a Bruker Optics FTIR Alpha spectrometer equipped with a DTGS detector and a KBr beamsplitter at 4 cm^−1^ resolution. Raman measurements were acquired using a Renishaw inVia Reflex Raman confocal microscope (Gloucestershire, UK) equipped with a diode laser emitting at 785 nm at a nominal power of 300 mW, and a Peltier-cooled CCD detector (−70°C) coupled to a Leica DM-2500 microscope. X-ray photoelectron spectroscopy (XPS) (K-ALPHA, Thermo Scientific SSTTI at University of Alicante) was used to analyze the surface of the samples. All spectra were collected using Al-K_α_ radiation (1486.6 eV), monochromatized by a twin crystal monochromator, yielding a focused X-ray spot with a diameter of 400 μm, at 3 mA *x* 12 kV. The alpha hemispherical analyzer was operated in the whole energy band, and 50 eV in a narrow scan to selectively measure the particular elements.

### Photoinduced water oxidation catalysis

Oxygen evolution experiments were performed in a 6.7 mL headspace Schlenk tube sealed with a rubber septum (PFTE). The Schlenk tube was covered with aluminum foil, in order to avoid an early light-induced reaction of the system, and filled with 1 mM (9.4 mg) [Ru(bpy)_3_]Cl_2_, 5 mM (14.9 mg) Na_2_S_2_O_8_, the desired amount of catalyst, and 12.5 mL of 40 mM KP_i_ buffer solution at pH 7.0. Experiments employing the **RuCo_9_** salt as catalyst were performed with and without addition of [Ru(bpy)_3_]Cl_2_, the former for comparison in the same conditions required for Co_3_O_4_ experiments. Suspensions were completely deaerated by purging with nitrogen. A baseline of 20 min was recorded to ensure that no oxygen leakage or side reactions took place. Next, the system was exposed to the light of a blue LED lamp (wavelength at peak emission = 465 nm; OSRAM Opto Semiconductors) working at 0.20 A and 11.4 V. The concentration of oxygen in the headspace was measured by employing a O_2_-sensor probe (Ocean Optics NeoFOX oxygen-sensing system equipped with a FOXY probe). Turnover number (TON) and turnover frequency (TOF) were estimated per **Co_9_** content as obtained from chemical analyses on fresh compounds (see SI).

## Results and discussion

### Visible-light-driven water oxidation by CsCo_9_ in heterogeneous conditions

**Scheme 1:**
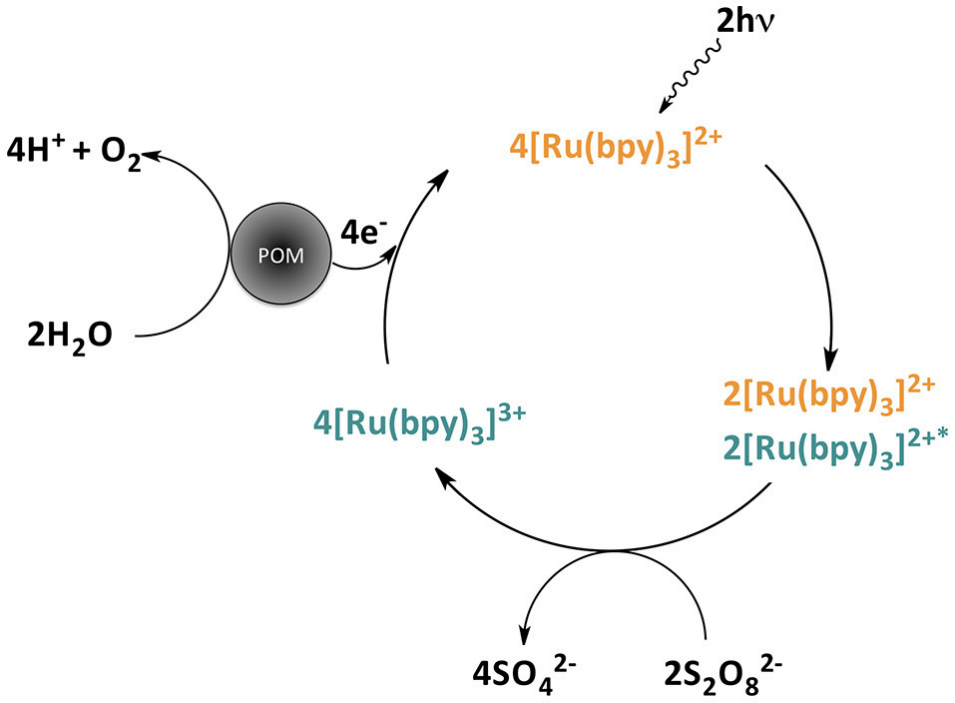
Schematic representation of the light-driven water oxidation catalysis reaction, employing [Ru(bpy)_3_]^2+^ as photosensitizer, S_2_${\text{O}}_{8}^{2-}$ as sacrificial electron acceptor, and a polyoxometalate as water oxidation catalyst (further side reactions giving rise to sulfate radical anions were omitted for clarity).

Water oxidation experiments were carried out with [Ru(bpy)_3_]^2+^ as a model photosensitizer and S_2_${\text{O}}_{8}^{2-}$ as sacrificial electron acceptor, in a suspension of the insoluble salt Cs_15_K[Co_9_(H_2_O)_6_(OH)_3_(HPO_4_)_2_(PW_9_O_34_)_3_] (**CsCo_9_**). Light irradiation (λ > 400 nm) of this mixture promotes oxygen evolution, which was monitored using a fluorescence O_2_-sensor probe for increasing amounts of **CsCo_9_** (1–50 mg). The proposed net reaction mechanism for light-driven water oxidation catalyzed by POMs is depicted in Scheme 1. No oxygen evolution was detected in the absence of any of the components.

**Figure 2:**
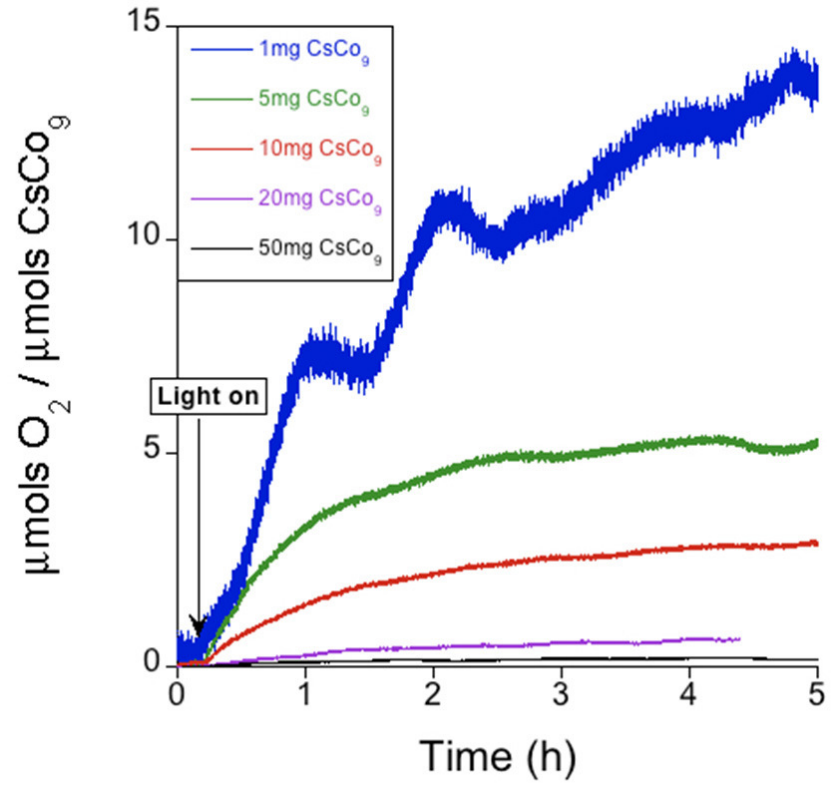
Oxygen evolution profile of the **CsCo_9_** salt in KPi (40 mM) buffer at pH 7, with [Ru(bpy)_3_]^2+^ (1 mM) and S_2_${\text{O}}_{8}^{2-}$ (5 mM).

The reaction starts with fast kinetics immediately after light irradiation, and slows down until oxygen evolution stops reaching a plateau after 2 h. We analyzed the oxygen production as a function of catalyst content (Figure 2 and Table 1). The highest values of turnover number (TON) and turnover frequency (TOF) obtained were 14.2 and 10.8 h^−1^, for the minimum quantity used (1 mg, ≈ 0.1 μmol). In terms of chemical yield (CY, see SI), a maximum 9.2% was reached for intermediate **CsCo_9_** contents (10 mg, ≈ 1 μmol) in the investigated range. After oxygen evolution, **CsCo_9_**was recovered from the reaction vessel to perform structural characterization. The FT-IR spectrum shows the typical **Co_9_** bands within the 1,100-400 cm^−1^ range, identical to those observed with the freshly made **CsCo_9_**. We also found additional bands in the region between 1,200 and 1,600 cm^−1^, which can be attributed to the bipyridyl (bpy) ligand (Figure S1). The same information is obtained from the Raman spectra (Figure S2). Moreover, comparison of the XPS spectra (Figure S3) showed the appearance of intense Ru peaks in the recovered **CsCo_9_**, and disappearance of the expected Cs peak (Figure S4). The data in their entirety suggest that cation exchange occurred under turnover conditions, i.e., Cs^+^ cations are replaced by [Ru(bpy)_3_]^2+^ cations. Indeed, the Raman spectrum of the recovered **CsCo_9_** is reminiscent of the corresponding Raman spectrum of the salt obtained by addition of an excess of [Ru(bpy)_3_]Cl_2_ to an aqueous K_16_**Co_9_** solution (Figure S9).

**Table 1:** Comparison of visible-light-driven oxygen evolution performance of **CsCo_9_** and **RuCo_9_** catalysts^(a)^.

**POM**	**Catalyst (mg)**	**1**	**5**	**10**	**20**	**50**
CsCo_9_ (first run)	TON	14.2	5.3	2.9	0.7	0.2
	TOF (h^−1^)	10.8	4.0	1.4	0.3	0.2
	CY (%)	4.4	8.4	9.2	4.0	3.6
RuCo_9_	TON	27.3	20.3	16.7	7.2	2.7
	TOF (h^−1^)	19.1	11.9	17.0	9.0	4.3
	CY (%)	7.6	28.4	47.6	42.4	36.4
POM	Catalyst (μm)	0.1	0.5	1	2	5

a *TON, total turnover number after completion of the reaction; TOF, slope of the oxygen evolution curve at the starting time; CY, total chemical yield after completion of the reaction*.

### Visible-light-driven water oxidation by RuCo_9_ in heterogeneous conditions

Addition of an excess of [Ru(bpy)_3_]Cl_2_ to an aqueous **KCo_9_** solution forms immediately an insoluble precipitate. The presence of the [Ru(bpy)_3_]^2+^ cation and the [Co_9_(H_2_O)_6_(OH)_3_(HPO_4_)_2_(PW_9_O_34_)_3_]^16−^ anion were confirmed by FT-IR spectroscopy (Figure S8) with the signature bands for both molecular species. However, the exact stoichiometry was difficult to completely assess. We carried out elemental CHN analyses, and metal ICP analyses, along with thermogravimetry analyses, and they were not fully consistent (Table S1). It is worthy to note at this point that our attempts to crystallize this compound in order to accurately characterize its composition and structure failed, because slow diffusion between solutions of cation and anion produce insoluble single crystals of the compound [Ru(bpy)_3_]_2_K_12_[Co_9_(H_2_O)_6_(OH)_3_(HPO_4_)_2_(PW_9_O_34_)_3_]·xH_2_O (Table S2, Figure S13). This Ru/POM stoichiometry is far too low in comparison with our **RuCo_9_** analyses (Table S1), and thus it is not representative of the **RuCo_9_** catalyst. The obtention of this crystalline phase, though, precludes the isolation of other salts with higher [Ru(bpy)_3_]^2+^ content, closer to the present **RuCo_9_** solid. The most plausible explanation is that **RuCo_9_** actually consists of a mixture of different [Ru(bpy)_3_]/**Co_9_** salts, and their slightly different solubility and composition gives small deviations depending on the given analytical technique. With all the analytical data taken into account (Table S1), we assign an average stoichiometry [Ru(bpy)_3_]_(5+x)_K_(6−2x)_[Co_9_(H_2_O)_6_(OH)_3_(HPO_4_)_2_(PW_9_O_34_)_3_]·(39+x)H_2_O (**RuCo_9_**), where −1 < x < 1 (see Table S1 and Figure S5). This powder is insoluble in water at room temperature with an average particle size of 374 nm (Figure S6).

**Figure 3:**
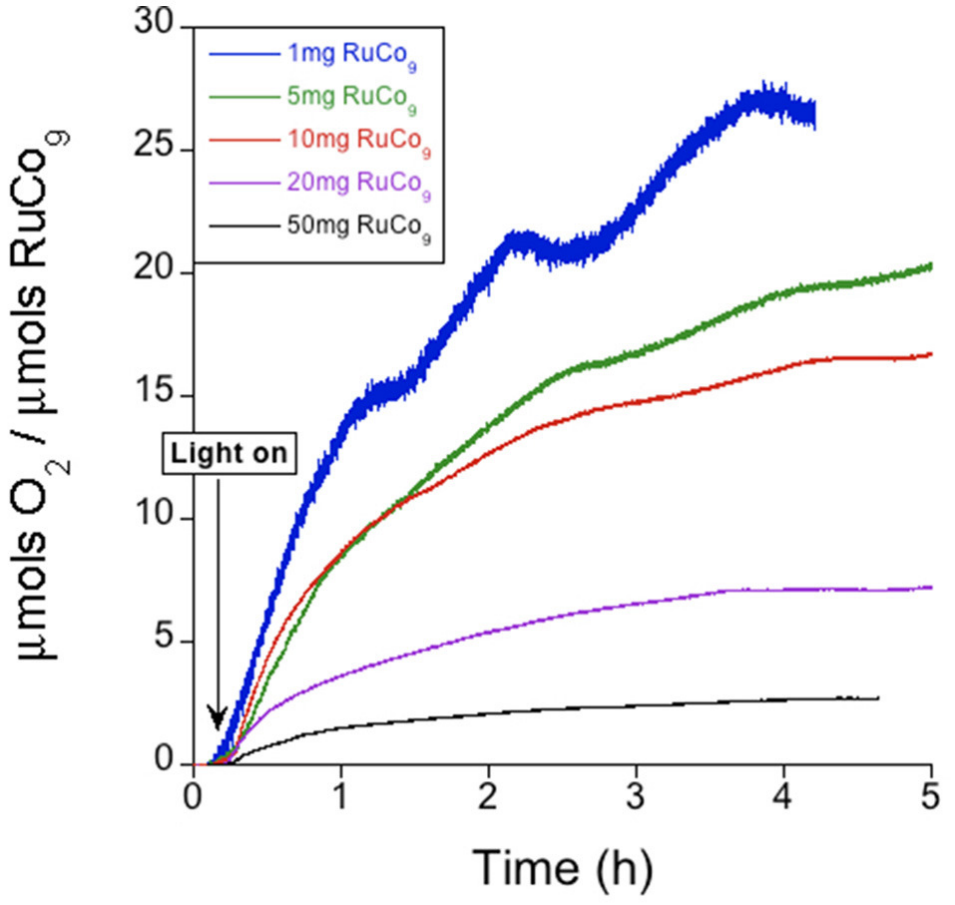
Oxygen evolution profile for solid **RuCo_9_** in KP_i_ (40 mM) buffer at pH 7 with S_2_${\text{O}}_{8}^{2-}$ (5 mM).

**Figure 4:**
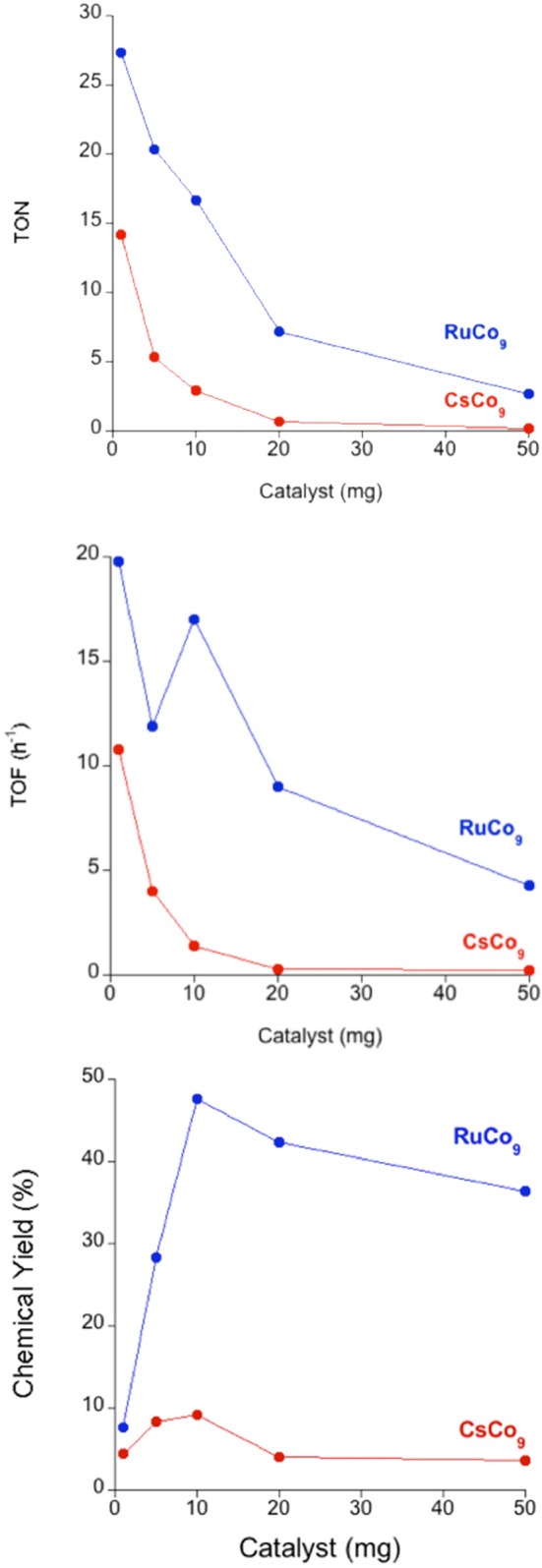
Comparison of TON, TOF, and chemical yield (CY) for **RuCo_9_** (blue) and **CsCo_9_** (red). Experiments were carried out in a KPi (40 mM) buffer at pH 7, with S_2_${\text{O}}_{8}^{2-}$ (5 mM). [Ru(bpy)_3_]^2+^ (1 mM) was added to the suspension of **CsCo_9_**, whereas no homogeneous photosensitizer was added for the **RuCo_9_** catalyst.

When a suspension of **RuCo_9_** in a solution of S_2_${\text{O}}_{8}^{2-}$ is irradiated (λ > 400 nm), oxygen evolution starts. In this case, the proposed reaction mechanism is analogous to that depicted in Scheme 1, but with photosensitizer and catalyst bound together in the solid state through electrostatic cation-anion interactions. Remarkably, the measured oxygen evolution in these conditions (Figure 3) is significantly superior to the first run starting from photosensitizer in solution (Table 1 and Figure 4). The maximum TON (27.3) and TOF (19.1 h^−1^) values are doubled, and the CY showed a remarkable increase up to 47.6%. Pulsed experiments confirmed that oxygen evolves exclusively when the light source is switched on (Figure S7).

### Stability of the RuCo_9_ system

**Figure 5:**
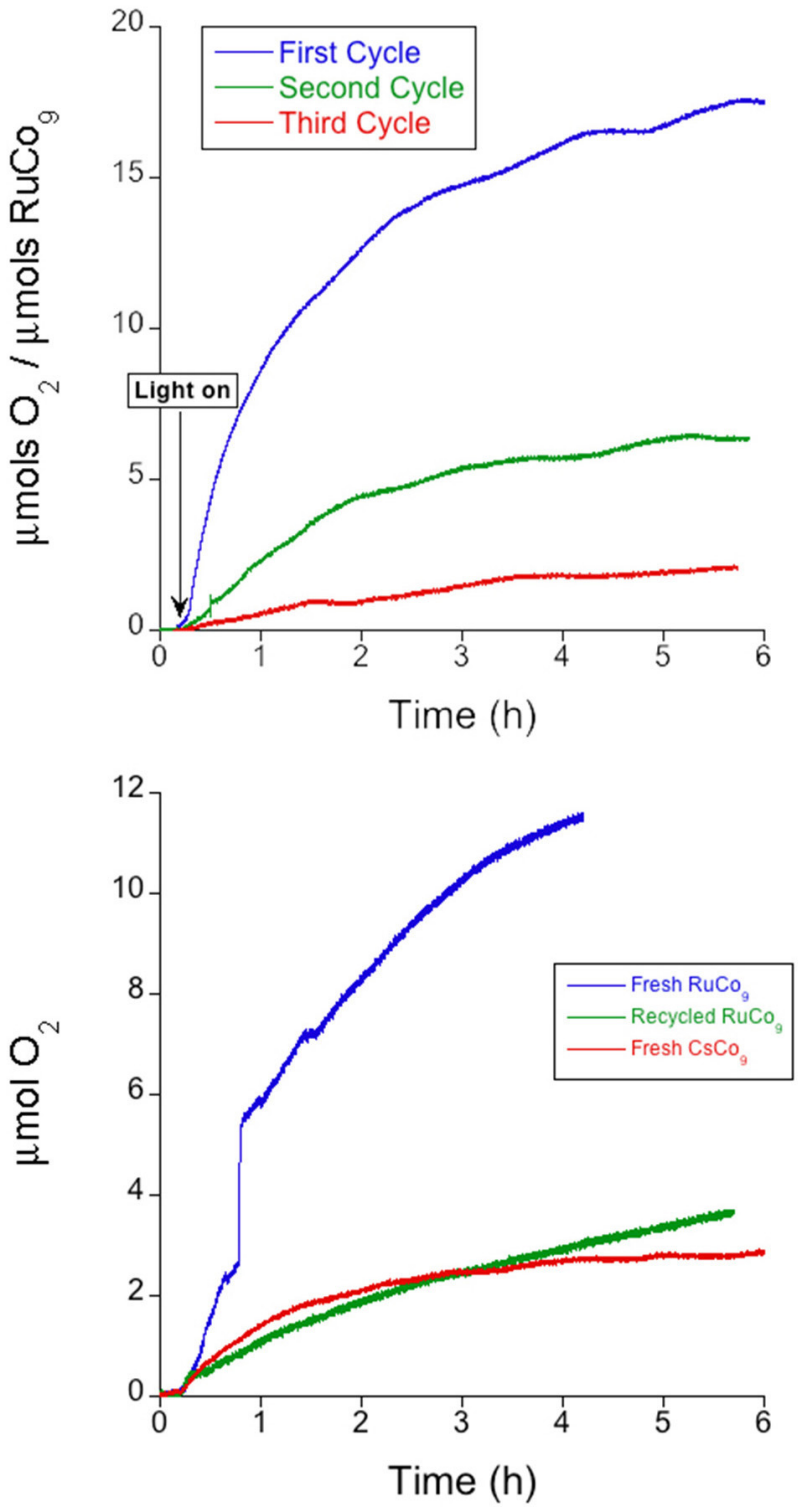
**Top:** Oxygen evolution with successive additions of oxidant S_2_${\text{O}}_{8}^{2-}$ (5 mM) to a KP_i_ (40 mM) buffered suspension of **RuCo_9_** (10 mg, 0.9 μmol) at pH 7. **Bottom:** Oxygen evolution after addition of photosensitizer [Ru(bpy)_3_]^2+^ (1 mM) and oxidant S_2_${\text{O}}_{8}^{2-}$ (5 mM) as solid reagents to a KP_i_ (40 mM) buffered suspension of recycled **RuCo_9_** at pH 7 compared with the same experiments starting from fresh **CsCo_9_**.

In order to determine the limiting agent in the photo-assisted oxygen evolution reaction, we carried out different tests. Successive additions of S_2_${\text{O}}_{8}^{2-}$ to the as-used **RuCo_9_** suspension indicate that oxygen evolution activity is severely affected after each cycle (Figure 5 and Table 2), i.e., the system can barely perform three cycles before reaching complete deactivation. After deactivation, addition of an aliquot containing the photosensitizer [Ru(bpy)_3_]^2+^ and S_2_${\text{O}}_{8}^{2-}$ to the reaction vessel restarts oxygen evolution, with rates and yields comparable to those obtained with **CsCo_9_** (Figure 5). This behavior can only be explained with deactivation of the photosensitizer in **RuCo_9_** recycling experiments, probably due to oxidative degradation of the organic ligands, during the harsh working conditions. The catalytic POM appears to be robust, since its performance is maintained during successive cycles.

**Table 2:** Comparison of the **RuCo_9_**-catalyzed light-driven oxygen evolution performance obtained for successive addition of S_2_${\text{O}}_{8}^{2-}$ (5 mM) to the reaction vessel^a^.

	**TON**	**TOF (h^−1^)**	**CY (%)**
1st cycle	16.7	17.0	47.6
2nd cycle	6.4	2.7	17.6
3rd cycle	2.1	0.7	5.6

a *TON, total turnover number at the final reaction time; TOF, slope of the oxygen evolution curve at the starting time; CY, total chemical yield at the final reaction time*.

### Analysis of adventitious CoO_x_ formation

In water oxidation with cobalt-based catalysts, it is fundamental to rule out the *in situ* formation of cobalt oxide CoO_x_, a competent heterogeneous WOC. This could occur through Co^2+^ leaching from the **RuCo_9_** salt, and the subsequent formation of CoO_x_ under oxidative conditions. Thus, we analyzed the as-used **RuCo_9_** with different experimental techniques in the search for traces of CoO_x_.

**RuCo_9_** was recovered from the reaction vessel after the visible-light-driven water oxidation experiments. The signature FT-IR and Raman bands of the **Co_9_** cluster remain identical when compared with pristine **RuCo_9_**, suggesting that the bulk POM structure is maintained during the experiments. Raman spectroscopy is particularly suited to detect even traces of CoO_x_ due to its high surface sensitivity, but no bands that could be assigned to a CoO_x_ species are present (Figures S8–S10).

As with fresh **RuCo_9_**, the elemental and ICP analyses showed small deviations, making difficult to confirm final stoichiometry. The numbers are not too different from the original stoichiometry (Table S1). However, we need to point out that these analyses show a decrease for all elements, except for W that increases. We assign this surprising result to the deterioration of the compounds during working conditions [triggered by the [Ru(bpy)_3_]^2+^ decomposition], making them even more insoluble, and untractable.

Another powerful surface-sensitive technique is XPS. Pristine and recovered **RuCo_9_** salts display analogous XPS spectra (Figure S11). The presence of CoO_x_ should include the appearance of a typical Co^3+^ peak below 780 eV (Chuang et al., 1976; Tan et al., 1991; Hara et al., 2000). Close analysis of the Co and O edges in search of such features that could be assigned to the presence of an CoO_x_ phase were negative. XPS spectra of **RuCo_9_** before and after oxygen evolution show intense bands only in the 780-783 eV range, which differ from those expected for CoO_x_ (Figure S12). This indicates that no cobalt oxide amounts are formed during turnover conditions within the detection limit of these techniques.

**Figure 6:**
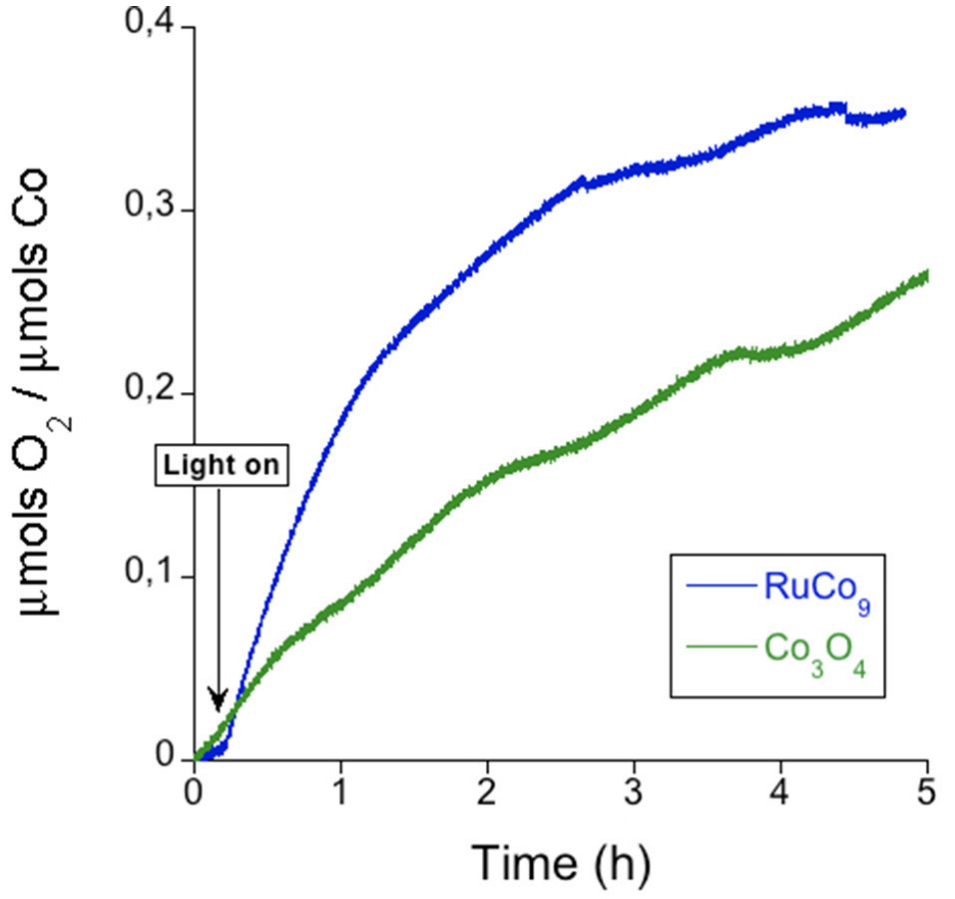
Comparison of the measured oxygen evolution employing equimolar Co amounts for **RuCo_9_** (blue) and Co_3_O_4_ (green). The experiments were performed in a KP_i_ (40 mM) buffer at pH 7 with [Ru(bpy)_3_]^2+^ (1 mM) as photosensitizer and S_2_${\text{O}}_{8}^{2-}$ (5 mM).

In order to gather additional indirect proof of the absence of the significant participation of cobalt oxide impurities, we compared the photo-induced oxygen evolution reaction starting from the **RuCo_9_** salt to the Co_3_O_4_ catalyst (Figure 6 and Table 3). For equimolar conditions, **RuCo_9_** displays an overall better performance, with faster onset kinetics and a higher efficiency. This is incompatible with the attribution of the catalytic activity observed for **RuCo_9_** to very small traces of CoO_x_, which may be below the detection limit of Raman or XPS techniques.

**Table 3:** Comparison of the light-driven oxygen evolution data catalyzed by **RuCo_9_** and by Co_3_O_4_ under the same reaction conditions^a,b^.

**Catalyst**	**TON**	**TOF (h^−1^)**	**CY (%)**
**RuCo_9_**	3.2	2.3	17.2
Co_3_O_4_	0.9	0.3	12.8

a *TON, total turnover number at the final reaction time; TOF, slope of the oxygen evolution curve at the starting time; CY, total chemical yield at the final reaction time*.

b *The experiments were performed in a KP_i_ (40 mM) buffer at pH 7 with [Ru(bpy)_3_]^2+^ (1 mM) as photosensitizer, S_2_${\text{O}}_{8}^{2-}$ (5 mM) as sacrificial electron acceptor, and with 13.99 μmols of Co in the form of **RuCo_9_** or Co_3_O_4_*.

## Conclusions

We compared the heterogeneous catalytic activity of two different **Co_9_** starting materials under visible-light-driven water oxidation conditions at neutral pH. Direct combination of **CsCo_9_** with a homogeneous photosensitizer yields a maximum turnover number (TON) of 14.2 and a maximum turnover frequency (TOF) of 10.8 h^−1^ with oxygen yields around 10%. Pre-catalytic incorporation of the cationic photosensitizer into the polyoxometalate salt through substitution of the alkali metal improves the oxygen evolution notably, affording chemical yields close to 50%. We associate this improvement to two beneficial effects of photosensitizer immobilization. On the one hand, the closer cation-anion (photosensitizer/catalyst) interaction in the solid state facilitates electron transfer, and therefore enhances the oxygen evolution kinetics. Additionally, the incorporation of the photosensitizer into the solid state partially improves its stability, an additional benefit to increase the efficiency of the overall process.

Our experimental data indicate that oxygen evolution eventually stops due to decomposition of the photosensitizer. Successive additions of photosensitizer re-start the water oxidation reaction at consistent rates, supporting the stable catalytic performance of **Co_9_**. We carried out careful surface analyses on the as-used catalyst in the search of traces of cobalt oxide. Neither Raman nor XPS spectroscopy showed any feature that could be associated with CoO_x_ species. Additionally, **RuCo_9_** exhibits superior catalytic performance than Co_3_O_4_. Thus, the hypothetical presence of undetectable CoO_x_ traces cannot be responsible for the observed catalytic activity. This supports the genuine catalytic activity of **RuCo_9_** for photo-induced water oxidation as the first example, to the best of our knowledge, of an effective photosensitizer/catalyst electron transfer in an ionic salt. The superior performance of this ionic composite opens up interesting perspectives for the use of such materials in the development of compact photoanodes for artificial photosynthesis.

## Author contributions

GP and JRG-M proposed the concept. GP, JRG-M, and JS-L designed the experiments. JS-L and FS carried out the experiments. All authors analyzed the data and contributed to the manuscript writing.

### Conflict of interest statement

The authors declare that the research was conducted in the absence of any commercial or financial relationships that could be construed as a potential conflict of interest.
