# Supplementary material for: Photoinduced Oxygen Evolution Catalysis Promoted by Polyoxometalate Salts of Cationic Photosensitizers
Source: Front Chem. 2018 Aug 14;6:302. doi: 10.3389/fchem.2018.00302 (PMC6102367; doi:10.3389/fchem.2018.00302)
Supplement: Supplementary file 1 [file Data_Sheet_1.pdf]

SUPPLEMENTARY INFORMATION

Table S1: CHN , ICP and TGA analysis for the **$RuCo_9$** salt before and after the light-driven water oxidation experiments, as compared with possible stoichiometries.

	%Ru	%Co	%W	%K	%C	%H	%N	%H2O
Fresh TGA								6.1
Fresh CHN					17.8	1.9	4.0	
Fresh ICP	3.9	4.2	45.5	2.5				
$[Ru(bpy)_3]_2K_{12}[Co_9].33H_2O$	2.1	5.4	50.5	4.8	7.4	1.2	1.8	6.1
$[Ru(bpy)_3]_3K_{10}[Co_9].35H_2O$	2.9	5.1	47.8	3.8	10.5	1.4	2.6	6.1
$[Ru(bpy)_3]_4K_8[Co_9].37H_2O$	3.7	4.8	45.4	2.8	13.3	1.6	3.3	6.1
$[Ru(bpy)_3]_{4.5}K_7[Co_9].38H_2O$	4.1	4.7	44.3	2.4	14.6	1.7	3.6	6.1
**$[Ru(bpy)_3]_5K_6[Co_9].39H_2O$**	4.4	4.6	43.2	2.0	15.8	1.7	3.9	6.1
$[Ru(bpy)_3]_6K_4[Co_9].41H_2O$	5.0	4.4	41.2	1.3	18.1	1.9	4.5	6.1
$[Ru(bpy)_3]_7K_2[Co_9].43H_2O$	5.6	4.2	39.4	0.6	20.2	2.0	5.0	6.2
$[Ru(bpy)_3]_8[Co_9].45H_2O$	6.2	4.0	37.7	0	22.1	2.2	5.5	6.2
Used TGA								4.0
Used CHN					15.9	1.43	3.8	
Used ICP	3.5	3.5	48.3	2.8				
$[Ru(bpy)_3]_2K_{12}[Co_9].21H_2O$	2.1	5.5	51.7	4.9	7.6	0.9	1.9	4.0
$[Ru(bpy)_3]_3K_{10}[Co_9].23H_2O$	3.0	5.2	48.8	3.9	10.7	1.2	2.7	4.1
$[Ru(bpy)_3]_4K_8[Co_9].24H_2O$	3.8	5.0	46.4	2.9	13.6	1.4	3.4	4.0
$[Ru(bpy)_3]_{4.5}K_7[Co_9].25H_2O$	4.1	4.8	45.2	2.5	14.9	1.5	3.7	4.0
$[Ru(bpy)_3]_5K_6[Co_9].25H_2O$	4.5	4.7	44.2	2.1	16.2	1.5	4.0	4.1
$[Ru(bpy)_3]_6K_4[Co_9].26H_2O$	5.2	4.5	42.2	1.3	18.5	1.7	4.6	4.0
$[Ru(bpy)_3]_7K_2[Co_9].27H_2O$	5.8	4.3	40.3	0.6	20.7	1.8	5.1	4.0
$[Ru(bpy)_3]_8[Co_9].28H_2O$	6.3	4.1	38.6	0	22.6	2.0	5.6	4.0

Table S2: Crystallographic parameters for the structure of $[Ru(bpy)_3]_2K_{12}[Co_9(H_2O)_6(OH)_3(HPO_4)_2(PW_9O_{34})_3]{\bullet}xH_2O$.

Crystal system	Triclinic
Space group	P–1
a(Å)	13.1889(8)
b(Å)	27.4351(7)
c(Å)	29.505(2)
α(°)	99.219(2)
β(°)	96.680(2)
γ(°)	93.043(2)
V (Å^3)	10438.7(3)
Z	2

The high disorder of the solvent molecules and counter-cations did not allow us to complete the data refinement. However, the large size of the $[Ru(bpy)_3]^{2+}$ allows us to confirm the $[Ru(bpy)_3]/[Co_9(H_2O)_6(OH)_3(HPO_4)_2(PW_9O_{34})_3]$ stoichiometry, since there is no available space for an additional $[Ru(bpy)_3]^{2+}$ in the unit cell (Figure S13). We assign a K_{12} stoichiometry because no other cations were used during synthesis.

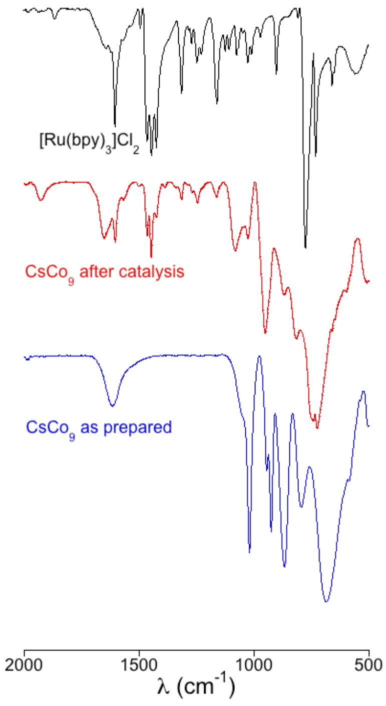

Figure S1: FT-IR spectra of the **CsCo$_9$** salt before (blue) and after (red) light-driven water oxidation experiments vs. commercial [Ru(bpy)$_3$]Cl$_2$ (black) as a reference.

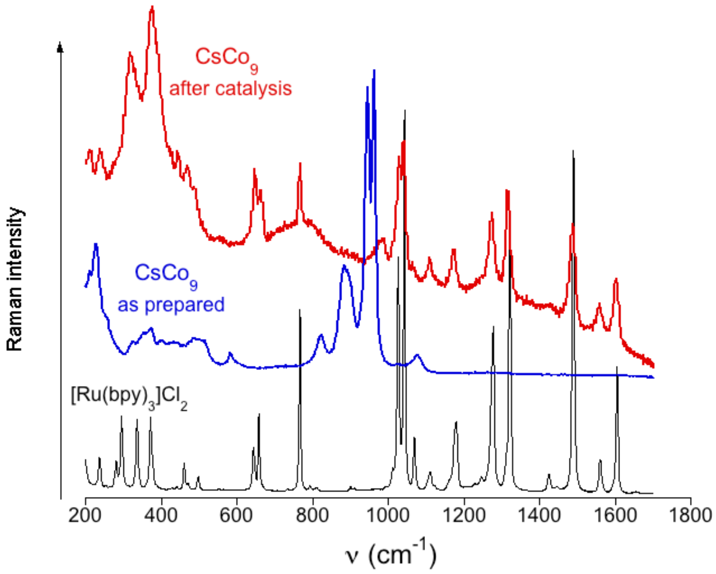

Figure S2: Raman spectra of the **CsCo$_9$** salt before (blue) and after (red) light-driven water oxidation experiments vs. commercial [Ru(bpy)$_3$]Cl$_2$ (black) as a reference.

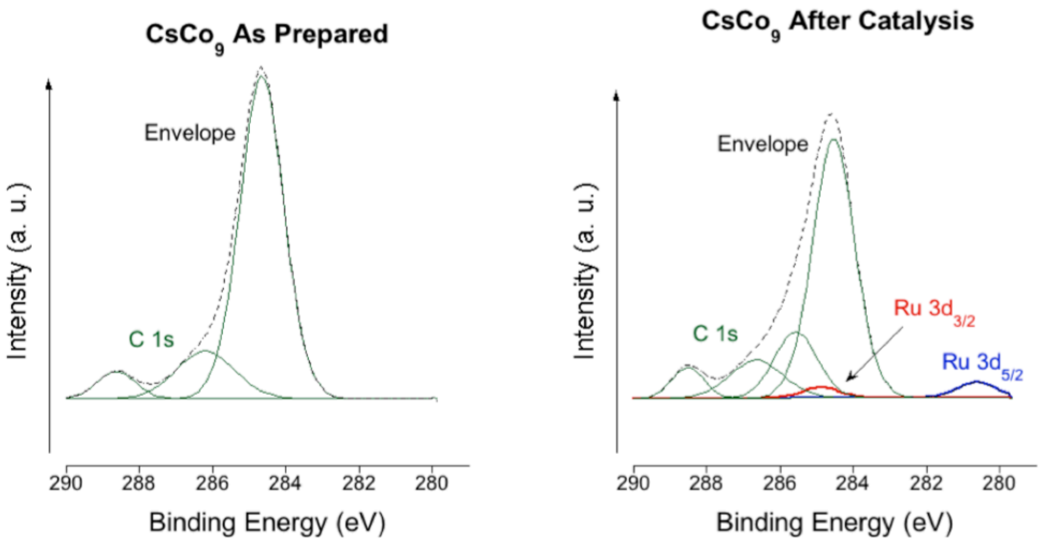

Figure S3: Ru 3d (blue and red) and C 1s (green) XPS spectra of **$CsCo_9$** as prepared (left) and collected after the light-driven water oxidation experiments (right).

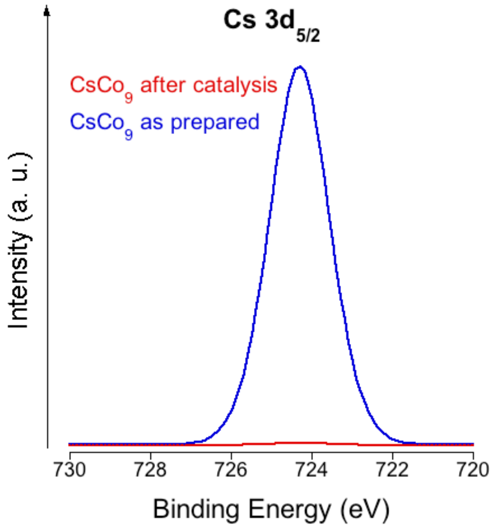

Figure S4: Cs $3d_{5/2}$ XPS spectra of **$CsCo_9$** as prepared (blue) and collected after the light-driven water oxidation experiments (red).

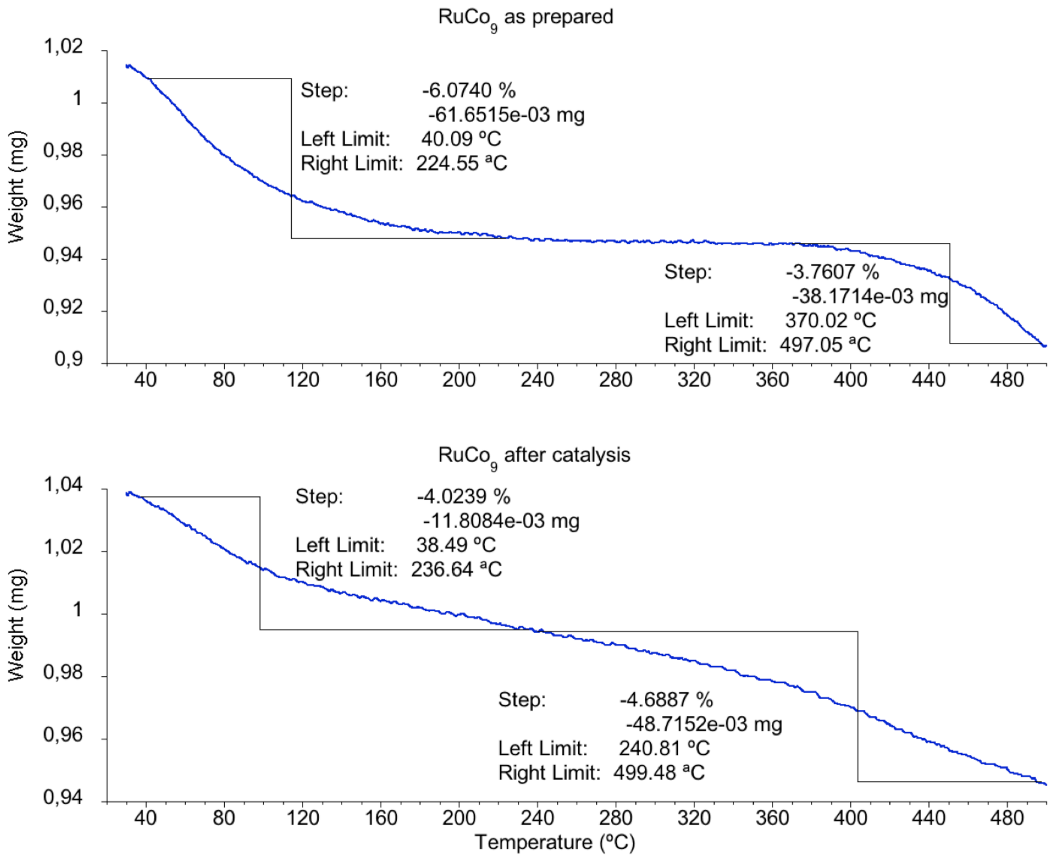

Figure S5: Thermogravimetric analysis of the **$RuCo_9$** salt before (top) and after (bottom) light-driven water oxidation experiments.

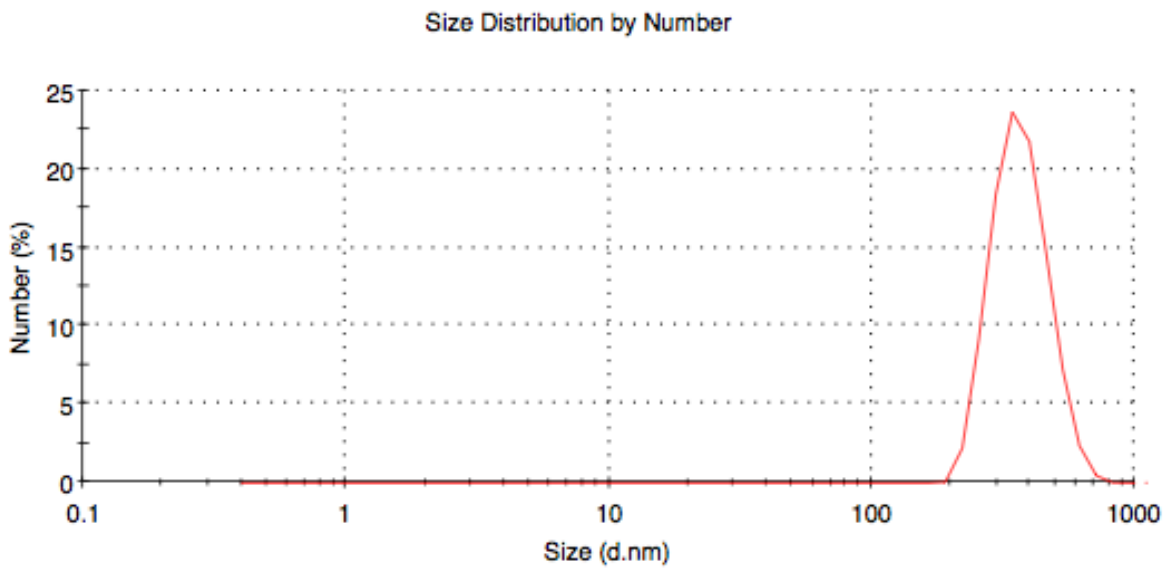

Figure S6: Dynamic light scattering measurement of as-prepared **$RuCo_9$** salt in suspension in water.

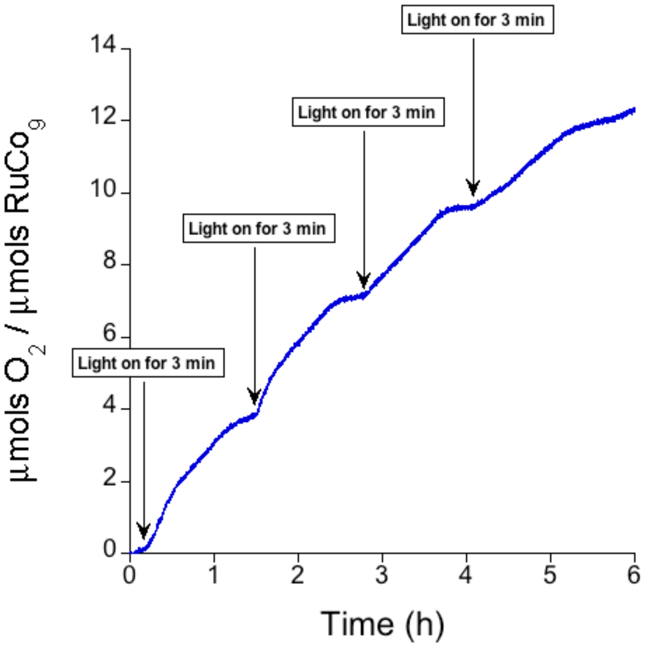

Figure S7: Measured oxygen evolution during the pulse experiments employing 10 mg of **RuCo₉** in a KP_i (40 mM) buffer at pH 7 with $S_2O_8^{2-}$ (5 mM) as sacrificial electron acceptor.

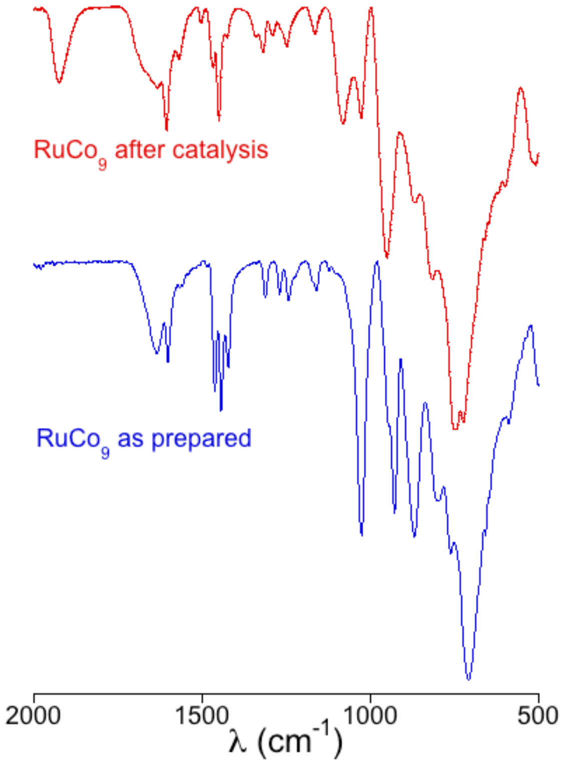

Figure S8: FT-IR spectra of the **RuCo₉** salt before (blue) and after (red) light-driven water oxidation experiments.

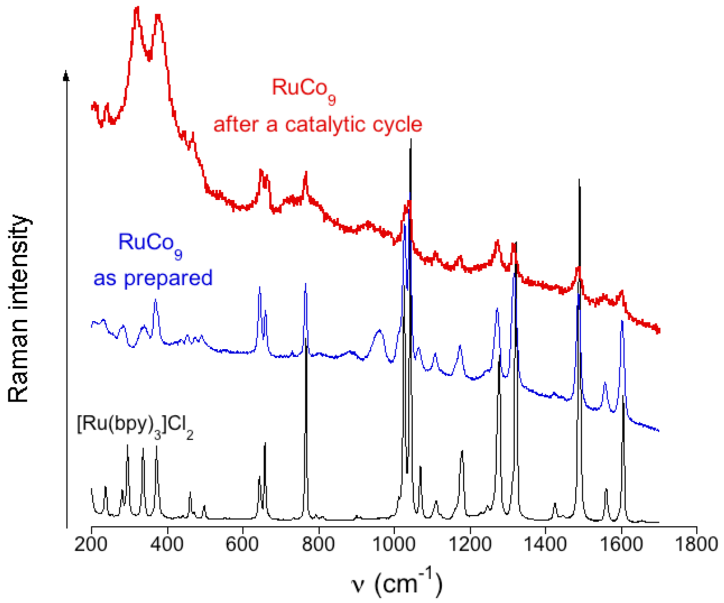

Figure S9: Raman spectra of the **RuCo9** salt before (blue) and after (red) light-driven water oxidation experiments vs. commercial [Ru(bpy)3]Cl2 (black) as a reference.

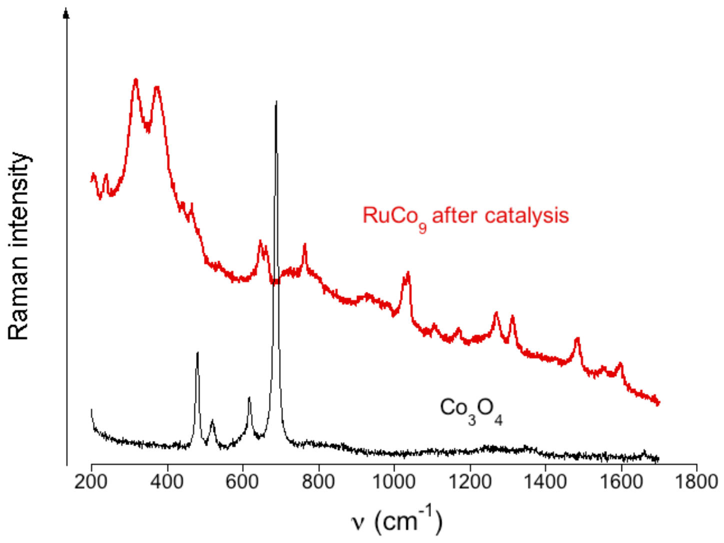

Figure S10: Raman spectra of the collected **RuCo9** salt (red) after light-driven water oxidation experiments compared to a pristine Co_3O_4 (black) reference.

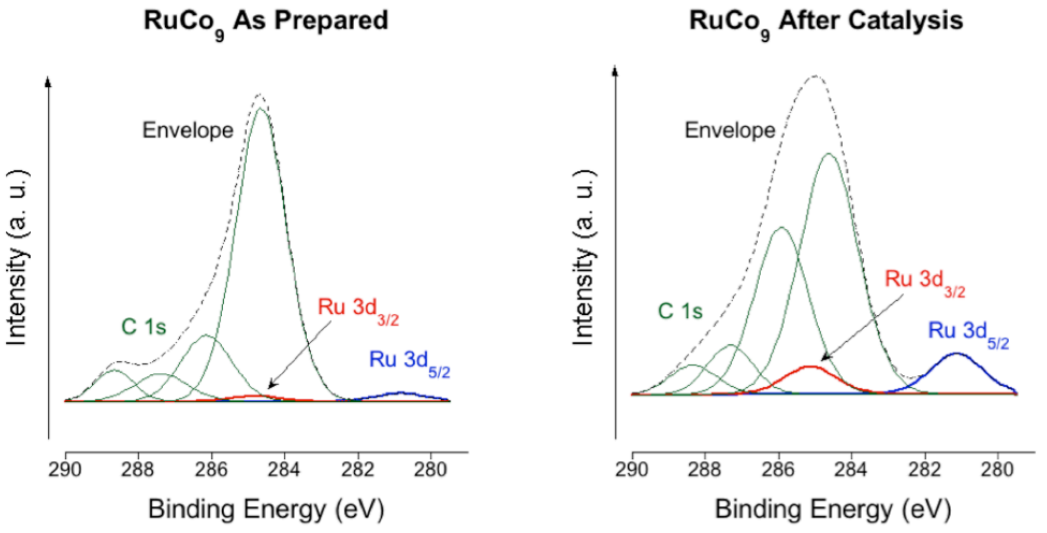

Figure S11: Ru 3d (blue and red) and C 1s (green) XPS spectra of **$RuCo_9$** as prepared (left) and collected after light-driven water oxidation experiments (right).

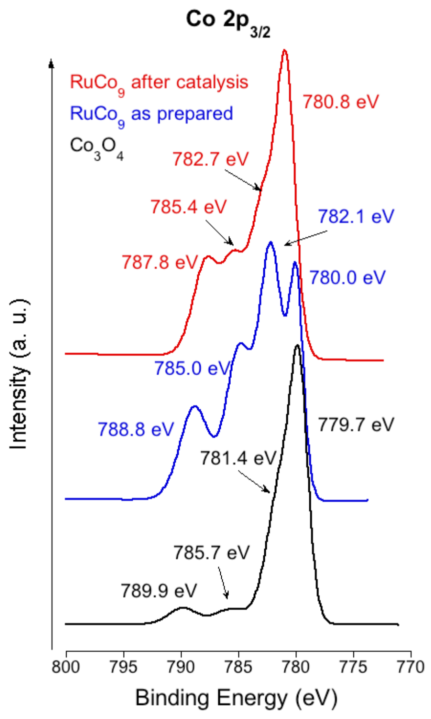

Figure S12: Co $2p_{3/2}$ XPS spectra of **$RuCo_9$** as prepared (blue) and collected after light-driven water oxidation experimens (red), compared to that obtained with pristine Co_3O_4 (black).

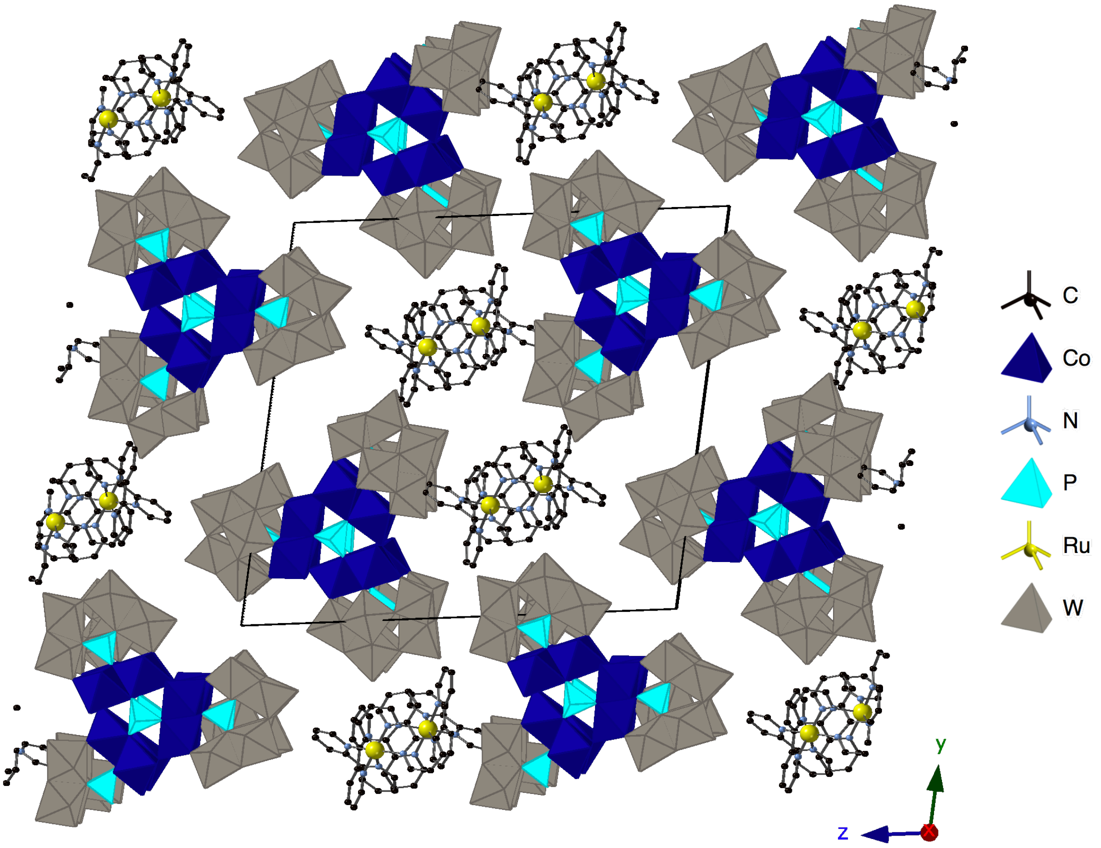

Figure S13: Representation of the unit cell for the single crystal of $[Ru(bpy)_3]_2K_{12}[Co_9(H_2O)_6(OH)_3(HPO_4)_2(PW_9O_{34})_3]\cdot xH_2O$ (cf. Table S1).

LIGHT-DRIVEN WATER OXIDATION CATALYSIS TESTS

The number of mols of O_2 produced during the experiments were calculated from the $\%O_2$ given by the oxygen-sensing probe employing the Dalton's law of partial pressures:

$$\%O_2 = \frac{P_{O_2}}{P_T} \times 100$$

where P_{O2} is the partial pressure of the O_2 in the gas-space, and P_T is the total pressure and equal to 1 atm. Therefore, by substituting P_{O2} into the ideal gas law we can calculate the number of mols of O_2 produced as:

$$n_{O_2} = \frac{\frac{\%O_2}{100} V_{gs}}{RT}$$

where n_{O2} = number of moles of O_2; $\%O_2$ = percentage of O_2 present in the gas–space; V_{gs} = gas–space volume (in L); R (gas constant) = 0.082 atmL/molK; and T = 298K.

The turnover number (TON) was calculated as:

$$\text{TON} = \frac{n_{O_2}}{n_{cat}}$$

where n_{O2} = number of moles of O_2, and n_{cat} = number of moles of catalyst.
The turnover frequency (TOF, h^{-1}) was calculated by plotting n_{O2}/n_{cat} vs. time. The slope at the beginning of the O_2 evolution follows a linear fit, and it is equal to the TOF, following the relationship:

$$\text{TOF} = \frac{n_{O_2}}{n_{cat} \cdot t} = \text{slope}$$

The chemical yield (CY, %) was calculated as:

$$\text{CY} = \frac{2 \cdot n_{O_2}}{n_{S_2O_8^{2-}}} \times 100$$

where n_{O2} = number of moles of O_2, and n_{S2O8} = number of moles of $Na_2S_2O_8$. The number two arises because the formation of O_2 requires the removal of four electrons, but the photochemical process using the $[Ru(bpy)_3]^{2+}/S_2O_8^{2-}$ assay accepts only two electrons.
